# Initiation and Continuation of Smoking in Iran: A Qualitative Content Analysis

**Published:** 2014-10

**Authors:** Hossein Ebrahimi, Mohammad Hasan Sahebihagh, Fazlollah Ghofranipour, Jafar Sadegh Tabrizi

**Affiliations:** 1 Department of Psychiatric Nursing, Faculty of Nursing and Midwifery, Tabriz University of Medical Sciences, Tabriz, Iran;; 2 Student Research Committee, Tabriz Health Service Management Research Centre, Department of Community Health Nursing, Faculty of Tabriz Nursing and Midwifery, Tabriz University of Medical Sciences, Tabriz, Iran;; 3 Department of Health Education, Tarbiat Modarres University, Tehran, Iran;; 4 Tabriz Health Service Management Research Centre, Department of Health Service Management, Faculty of Management and Medical Informatics, Tabriz University of Medical Sciences, Tabriz, Iran

**Keywords:** Iran, Predisposing Factors, Qualitative Research, Smoking

## Abstract

**Background:** Smoking is the cause for many preventable deaths worldwide. The rate of smoking has not increased in Iran in the past two decades, but its increase among adolescents and young adults is a concern. This study investigates the risk factors of initiation and continuation of smoking in Iran using a qualitative approach.

**Methods:** This is a qualitative content analysis study conducted on 12 smokers and 6 non-smokers in 4 selected cities in Iran. Data were collected with deep and semi-structured interviews, verbatim transcription and simultaneously coding. Then, they were analyzed through content analysis.

**Results**: Three themes and 16 subcategories emerged. The themes were personal inefficacy with 6 subgroups included inadequate information, low age, curiosity, consideration of smoking not as a major problem, wrong beliefs, and making reasons. Family inefficacy with 4 subgroups included poor authority, lack of reaction, existence of stressors, and history of smoking. Vulnerable social environment with 6 subgroups included poverty, social stressors, magnification of smoking, network of cigarette smoking, smoking as a norm and convenience of access.

**Conclusion:** Recognition of smoking among children, modification of wrong beliefs about smoking, empowerment of the individuals against smoking from the very childhood, consideration of familial stress and crisis, and ultimately, paying attention to the role of social variables will play a major role in prevention of smoking and encouraging individuals to quit smoking.

## Introduction

Smoking, especially cigarette smoking, leads to many preventable deaths all over the world.^1^It is estimated that the number of smokers increases from 1.3 billion to 1.6 billion people in 2025. Its associated mortality is estimated to increase to 8.3 million persons in 2030 from 4.8 million persons in 2006.^2^ In Iran, the rate of smoking has not increased in the past two decades, but with regard to warning reports of smoking among adolescents and young adults, this subject is of great concern in this age group.^3^

There are often numerous factors for initiation and continuation of smoking, and researchers have tried to detect these factors in various contexts. Most of the research, conducted in this field is quantitative research. A review of previous studies shows that low age,^2^^,^^4^^-^^6^ male gender,^6^^-^^9^ low education status,^2^^,^^10^ lack of knowledge about smoking and its side effects,^11^^,^^12^ low economic status,^2^^,^^6^^,^^10^^,^^13^^-^^15^ familial conflicts and disintegration,^2^^,^^5^^,^^6^^,^^16^^,^^17^ friends and peers who smoke,^6^^,^^7^^,^^18^^,^^19^ smoking history in the family,^3^^,^^5^^,^^6^^,^^8^^,^^9^^,^^11^^,^^17^ living alone and far from the family,^6^^,^^9^^,^^16^^,^^19^ and magnification of smoking,^20^^-^^24^ are risk factors for initiation and continuation of smoking.

In addition to the above-mentioned risk factors that were pointed in many studies, some studies have reported some other risk factors such as mothers’ depression,^5^ having a disability,^25^ low self-esteem, high temptation for trying a cigarette,^9^ positive attitude and thoughts about smoking, being intended to smoke,^16^^,^^26^ exposure to the people smoking in public places, free access to cigarettes,^8^ having a smoking teacher,^11^ initiation of smoking with menthol containing cigarettes,^27^ seeking for a stimulating substance, smoking for fun,^19^ smoking electronic cigarettes,^28^ reduction of nicotine in cigarettes and lowering it to a non- addictive level,^29^ joy, imitation, show-off and curiosity,^7^ listening to music,^30^ and perceived social advantages for the smokers like looking relaxed, feeling peaceful, looking like a normal person, and feeling grown up.^12^

Researchers believe that although the aforementioned studies have detected a high number of risk factors for smoking, the experiences of smokers in the initiation and continuation of smoking have not yet been investigated. To access a holistic list of predisposing factors, it is better to listen to smokers’ remarks about their initiation and continuation of smoking through a qualitative approach. Qualitative studies identify the background and condition of initiation and continuation of smoking through precise analysis of smokers’ remarks. Hay (2005) believed that current strategies to measure perception of smoking risk factors are in numeric estimations, and cannot adequately measure individuals’ feelings and thoughts concerning smoking risk measurement.^31^ Qualitative research introduces the real structure of smoking in the individuals’ world^32^ and enhances the understanding of researchers in health sciences about smoking and enables them to use their knowledge while working with the clients, giving health care and through general fields of smoking control.^33^ Considering the above-mentioned issues, we aimed to detect smokers’ experiences concerning the initiation and continuation of smoking in a qualitative approach.

## Materials and Methods

The present study was conducted through a qualitative research in a conventional content analysis. The goal of conventional content analysis is description of the phenomenon, and it is employed when the resources about a phenomenon are low. In conventional content analysis the code categories are directly extracted from the interview transcripts.^34^

The participants comprised 12 smokers and 6 non-smokers. Of whom 13 were male and 5 were female. Their age ranged from 21 to 51 years and their education varied from primary school to master’s degree. With respect to their occupation, two were homemakers, four workers, nine employees, two were university students and one was self-employed. Seven participants resided in Tabriz, two in Tehran, seven in Ilam and two resided in Boushehr (Based on the finding of Mehrabi’s study Ilam and Boushehr have lowest and  highest prevalence of smoking respectively).^35^ Mean score of Fagestrom test (smoking dependency measurement test) of the smoking participants was 8.33.

Primary participants were selected from familiar smokers. Interviews were conducted in public gardens, at participants’ work place, the researcher’s office, participants’ house or researcher’s house.  Interviews were made with 16 participants and two participants wrote down the story of their smoking as a scenario and handed them to the interviewer. Interviews were conducted by non-smoking participants for testing rival experiences in background of initiation and continuation of smoking. All interviews were conducted in 2012.

Participants underwent deep and semi-structured interviews. At the beginning of the interviews, research goals and method were explained to the participants. Interviews started by some general questions. The question, asked from the smokers was “how did you start smoking?” please tell about your story of smoking from the first day to present”. Non– smoking participants were also asked “have you ever been offered a cigarette to smoke?”, “Have you ever been in constant touch with a smoker?”, “please tell me the story of some interactions you have had with smokers”. Maximum duration for interviews was one hour, two of the participants’ story did not end within one hour, and therefore another interview was arranged. 

In present study we used Graneheim and Lundman method for data analysis.^36^Every interview was selected as a unit of analysis. The words, sentences, or paragraphs with a specific mean were defined as coding unit or meaning unit, concepts of meaning unit were distillated in 4-5 word and it was abstracted in a code. The codes with similar concepts created categories.

To reach trustworthiness concerning credibility, the researcher had a long and close acquaintance with the participants (for two years). The researcher had research projects and needed working experience in the context of smoking. Participants were selected from a vast spectrum (men, women, young, old, light and heavy smokers, and various locations in Iran), Transcripts and scenarios including the extracted codes were given to the participants and were confirmed by them. The consistency of the codes with the categories and concepts was checked and confirmed by two of the colleagues who were experts in qualitative research. Regarding dependability, interviews transcripts were handed to the researcher’s co-worker, and after he coded the transcripts, consistency of the codes was checked and confirmed. For confirmability of the study, sampling mode, questions development and interview tool, the method of coding and category extraction and modification were recorded and documented. To achieve the upmost transferability, the findings of the study were handed to a smoker who did not participate in the study and were confirmed by him concerning their truthfulness and consistency with his own experiences.

This project was approved by ethics committee of research and technology vice-chancellery of Tabriz University of Medical Sciences. All the participants signed a written consent form at the beginning of the interview. 

## Results

Three themes and 16 subcategories emerged. The themes were personal inefficacy with 6 subcategories, family inefficacy with 4 subcategories, and vulnerable social environment with 6 subcategories (table 1).

**Table 1:** Themes, Sub-categories and codes of initiation and continuation of smoking

**Theme**	**Sub-category**	**Codes**
Personal inefficacy	Inadequate information about smoking	Knowledge deficit about: nature of smoking, harms of smoking, consequences of smoking. Lack of experience: no prior experience about smoking, no smoking member in the family, Few smokers in the kin network.
	Low age	Experience of smoking in childhood. Beginning of smoking in adolescence.
	Curiosity about smoking	What is the taste of cigarettes? What will happen in my body when I smoke?
	Consideration of smoking not as a major problem	Smoking is a kind of fun, *one puff was not a problem*, we easily quit whenever we liked, smoker is a person that smoke least 100 stick, and therefore we aren’t formal smoker.
	Wrong beliefs about smoking	Usefulness of smoking, exaggeration about smoking harms, neutralizing the side effects of smoking, shallow breath for smoking, difficulty of cessation, the feeling of low worth of health.
	Making excuse	Ignoring of self smoking, Emphasizing their capabilities.
Family inefficacy	Poor family head authority	Low training, low supervision, low physical abilities, low mental abilities, low managerial abilities.
	Existence of stressors and crisis in the family	Feeling of loneliness, existence of conflicts and disputes in the family, history of parents separation and divorce, death of both parents or any of them, and existence of happy events like a childbirth.
	Lack of reaction	Ignoring the smoking behavior in the family, confirmed smoker behaviors.
	History of smoking in the family	Non-smokers parents, brothers’ smokers, sisters’ smokers.
Vulnerable social environment	Poverty	Parents’ illiteracy or low literacy, low economic, poor environment, shortage of recreational and sports facilities, shortage of cultural facilities.
	Social stressors	War, inflation, prices change, joblessness and missing a job.
	Magnification of smoking	Direct magnification: size, shape, color and design of cigarette pocket in shops. Smokers’ behaviors, smoking at forbidden places. Indirect magnification: films in TV and cinemas.
	Network of cigarettes smoking	Neighborhood friends, neighborhood peers, school friends, school peers, sports friends, sport peers, Family members included sisters, brothers and parents.
	Smoking as a norm	Smoking as a sign of: growth, passing adolescence period, entering the society and socialization. smoking among physicians, nurses, health professionals, university teachers, teachers and policy makers.
	Convenience of access	Accessible from supermarkets, kiosks, shops, salesman crossroads. Accessible for children, adolescences, youth, adults and older people. Accessible for men and women.

*Personal Inefficacy*

Personal inefficacy is the existence of characteristics that inhibit correct decision making for having a safe life. In this situation a person may select high risk behaviours like smoking. In this study some characteristics with respect to smoking included inadequate knowledge, low age, curiosity in smoking, considering smoking as a not do major problem, wrong beliefs, and making excuses. We will explain each subcategory in the following:

*1) Inadequate information about smoking: *Most of the participants had inadequate information about cigarettes and their side effects. Participant 5 said:**

*“I wish, when I started smoking, someone would warn me about that. I was so young and knew nothing about cigarettes and their harm. In fact, now, I am wise enough”.*

*2) Low age: *Most of the participants started smoking under 18 years of age and many had experienced smoking in childhood.

*3) Curiosity about smoking: *Smokers often wanted to know what a cigarette and its taste was like when they had started smoking. Participant 6 said:

*“My mom’s aunt used to smoke. At the age of 5-6 years, me and my cousin stole a cigarette and smoked it somewhere to see what it was”*.

4) *Considering smoking as a not so major problem*: Some believed smoking was fun thing and not a major problem when they started smoking. They believed they could smoke and easily quit whenever they liked. Participant 7 say:**

*“That time I thought one puff was not a problem. I would test it, but, then after I found out I was attracted to cigarettes”.*

*5) Wrong beliefs about smoking:* Wrong beliefs, in the present study, refer to those beliefs which result in smoking. One of these beliefs was “usefulness of smoking”. Some believed that if cigarette is appropriately smoked, it not only does not harm the body but it is also beneficial for the body since it is refreshing. Participant 2 said:**

*“If you are healthy and your mind is happy and you smoke a stick after each meal, it has lots of benefits for the body. The pleasure of smoking a cigarette after a meal, after drinking a cup of tea can never be found in anytime else*.

Another wrong belief was exaggeration about the harms of smoking. They believed that smoking is not as harmful to the health as it is said to be. These people introduce healthy smokers and non-smokers who were sick as examples to prove their beliefs. Participant 2 said: 

*“I see those at my age. There is no difference between my health and theirs. I have friends who despite not smoking and being at my age, have a worse condition”.*

Another belief was “neutralizing the side effects of smoking”. They believed that complications of smoking can be neutralized through sport and a rich nutrition. Participant 2 said:

*“I go mountain climbing, play sports, and try to keep my arteries open despite smoking. I try to have open arteries”.*

Another belief is “shallow breath for smoking”. They believed that shallow breath for smoking and not entering the smoke into the lungs is safe and prevents smoking complications. Participant 1 says: 

*“Smoking is different person to person; my cigarette (smoke) does not go as down as here (showing his vocal cord)”.*

Another belief was “difficult cessation”. The believers were hopeless that they could quit one day, and therefore, continued their smoking. Participant five said: 

*“In fact, when cigarette (nicotine) enters you vessels, it is hard to quit, I already missed two of my children because of smoking. Although I was terrified, I could not quit, I swore not to smoke, but could not quit”.*

Another belief was “the feeling of low value of health”. Value of health was low in the smokers’ minds, therefore they tried less to maintain their health For instance, participant one said:

*“You see, they tell me, you will die if you smoke, and I answer, I do not mind.”*

6) *Making excuses*: Some participants made excuses for their smoking, which were not related to the subject. For instance, participant one making reasons for his smoking said:

*“A friend who had quitted smoking was proud of that. I told him, your intention is so strong in cessation, mine is also strong in mountain climbing. These comparisons can convince one to smoke.”*

*Family Inefficacy*

Family inefficacy is defined as the existence of characteristics in the structure and function of the family that inhibit correct decision making for having a safer life.  In this situation one or more family members may select high risk behaviors like smoking. In this study some characteristics revealed in relation to smoking included poor family head authority, existence of stressors and crisis in the family, creating overt feelings of satisfaction, and history of smoking in the family. We will explain each subcategory in the following:

*1) Poor family head authority*: Most of the smokers had either lost the head of the family or had been deprived from enough training and supervision due to low physical, mental, and managerial abilities of the family head. Participant eight says:

*“I should also say my family ties were not so tight, my parents were somehow busy and these reasons lead to smoking and addiction”.*

*2) Existence of stressors and crisis in the family*: Based on smokers’ remarks, being far from the family, living alone and far from the family, the feeling of loneliness, existence of numerous conflicts and disputes in the family, history of parents separation and divorce, death of both parents, or any of them, and existence of happy but stressful events like a childbirth, children’s marriage, and win or defeat of the favourite soccer team  play a role in initiation and continuation of smoking among smokers. Participant one said:

*“As far a, I can remember, my father was an illiterate person, could not hear well and was naïve. When my mother divorced, he did not have enough authority to open up the subject and firmly say smoking is harmful”.*

3) *Lack of reaction*: In some cases, family members especially parents may ignore risky behaviors of their children such as smoking. Participant six said:

*“I cannot say my parents did not know it (my smoking), they knew it, but as they ignored it, I continued stealthy smoking.”*

4) *History of smoking in the family*: Based on our obtained findings, parental smoking plays a protective role while brothers’ or sisters’ smoking acts as a facilitator in adolescents’ smoking. Participant four said:

*“I started smoking when my brother and I used to work in a shop. My brother saw me smoking one day and asked me if I was used to smoking, and I answered, occasionally. He said if occasionally, no matter, smoke!”*

*Vulnerable Social Environment*

It is a susceptible environment that grows one or more threatening factors for health. In this situation the health of residents may be at risk. Examples of threatening factors for growing unsafe behavior like smoking in this study included poverty, social stressors, magnification of smoking, network of cigarette smoking, smoking as a norm, and convenience of access. We will explain each subcategory, as follows:

*1) Poverty*: Poverty played was influential in the initiation and continuation of smoking in both economic and cultural contexts. Most of the participants associated their smoking to living in a low income and poor family, shortage of recreational and sports facilities, shortage of cultural facilities and their parents’ illiteracy or low literacy. Participant one said:

*“We were poor, when I went to school in the morning, I worked in the afternoon. When I went to school in the afternoon, I worked in the morning.”*

*2) Social stressors*: Based on the participants’ experiences, social pressures like war, inflation, price changes, joblessness and missing a job played a role in their initiation and continuation of smoking. Participant two said:

*“Following cease-fire (1988), suddenly price of gold went down, I had bought a gram of gold for 65000 Rials (*Currencies* of Iran)  , after cease-fire price of gold downed to 15000 Rials,  I was under too much stress, so I started smoking again.”*

3) *Magnification of smoking*: Magnification of smoking played a major role in smoking. Participant one said*:*

*“With so much magnification of smoking (in the mass media), we think of it, believe me, if cigarette were not so magnified (in the mass media), I would quit very easily”.*

4) *Network of cigarette smoking: *this network included friends, peers, family members and people that act as a model for children and adolescences in smoking. Children and adolescents’ friends and their peers played a key role in their smoking. Smoker participant three said:

*“A friend plays a key role in one’s smoking. One friend may tell you men do not smoke, it is harmful, another may buy you a single cigarette and give it to you to smoke.”*

Our findings showed smoking sibling and friends had similar roles in the initiation of smoking, but the smoking fathers’ role were different. Some smoker participants initiated smoking by following their older brothers as role modelling, whereas their fathers did not have any role in the initiation of their smoking. Non-smoker participant 12 said:

*“The main reason I didn’t go towards smoking was my father, I saw his problems, therefore I didn’t let myself be tempted to smoke.”*

*5) Smoking as a norm*: Smoking as a sign of growth, as a sign of passing adolescence period, as a sign of entering the society and socialization, or in the other words, the position of cigarette smoking in the social life, and smoking among physicians, nurses, health professionals, university teachers, teachers and policy makers all played a major role in normalization of smoking in the society. Participant one said:

*“Doctors are liars, if they are right, why they themselves smoke. If they were right, they would respect their own words.”*

*6) Convenience of access*: Based on the participants’ experiences convenient access to cigarettes and existence of no limitations in selling cigarettes played a role in the initiation and continuation of smoking. Participant one said:

*“When I was a child, I worked with my brother, our car broke down, and my brother is looking for a repairman, I went to the village for buying bread, I didn’t buy bread, and therefore I went to the supermarket for buying biscuits, I saw a cigarette and was tempted, and I bought a biscuit and two packets of cigarettes, so I started smoking.”*

## Discussion

This study aimed to assess the experiences of smokers about initiation and continuation of smoking in Iran through a qualitative approach. An analytic approach to the findings showed that in some cases, the findings of the present study are consistent with previous studies. For instance, poor knowledge about cigarettes,^11^^,^^12^^,^^37^ low social and economic class,^2,6,10,13-15,37 ^curiosity,^7^ smoking for fun and considering it as a minor issue,^19^ magnification of smoking and the effect of friends and peers network,^6,9,18,19,37-41^ and convenience of access to cigarettes^8^^,^^42^^,^^43^ were among the issues pointed out in previous studies. In a qualitative research, Niknami et.al (2008) found that poor knowledge, wrong beliefs, frustration, curiosity, cheap price of cigarettes, easy access to cigarette, media influences and role of peer groups were influential.^44^ The finding of our study were similar in some themes and sub-categories and yet differ from some others. It seems that the existence of differences between findings of qualitative studies is a normal phenomenon; this is an important reason for study repetitions.

Some of the findings of the present study had already been discussed in previous studies, but they were different in quality. Among these findings, there were low age, family history of smoking, stress in the family environment and consideration of smoking as a norm. The age of smoking initiation was reported adolescence in most of the previous studies. Our obtained findings showed that most of the participants experienced smoking in childhood, and then smoking was suspended for a period. Next, it was started in adolescence and probably led to formal smoking. Based on the findings of the present study, activation of smoking preventive programs from adolescence is too late, and education on the harms of smoking and its negative effects on health should start from childhood and in form of empowerment programs. 

In previous studies on the history of smoking at home,^8^^,^^9^ existence of a smoker in the family,^6^ existence of a smoking brother or sister, existence of more than one smoker in the family,^17^ a smoking mother,^5^ and a smoking father^3^^,^^8^^,^^11^^,^^17^^,^^39^ have been reported as the risk factors for adolescents’ smoking initiation. In a recent qualitative study,  smoking of fathers was introduced protective as a factor against smoking.^45^ Our interviews with non-smokers showed that existence of a smoking father in the family not only is not a risk factor but also acts as a protective factor as the adolescents are less attracted to cigarettes when they see its complications in their father. Most of the smokers had a non-smoking father. Those smokers with smoking fathers claimed that their fathers were not role models in their smoking, and their smoking friends, peers, brothers and sisters were the role models.

In previous studies, living alone,^9^ not living with parents,^6^ living in nursing homes,^16^ and absence of adults,^19^ family disrupts and disintegration^2^^,^^5^^,^^6^^,^^16^^,^^17^ were reported as the risk factors for initiation and continuation of smoking. There is no doubt that these can be the major factors for existence of stress in the family. We found that in addition to the above–mentioned risk factors, even happy events like childbirth, children’s marriage and win or defeat of the favourite soccer team can be stressful and increase smoking.

In a few previous studies “perceived social benefits of cigarettes” like looking calm, feeling peaceful, looking like a common person and feelings of being grown have been reported as the risk factors for initiation and continuation of smoking.^12^ If this advantage is accepted as a normalization factor in the society, the findings of the present study can add other factors to this context, which play a major role in normalization of smoking in the society such as cigarette smoking among policy makers, famous people, physicians, nurses and other health providing staff. Our interviews with smokers showed that these people play a key role in initiation of smoking and put the cessation encouragement programs in serious trouble. Findings of Arora (2013) in a qualitative study showed active involvement of local policy makers and health professionals are important in creating and reinforcing tobacco-free norms.^37^

Some of our obtained findings were not observed in previous studies of other researchers including “wrong beliefs about smoking”, “lack of reaction”, “making excuse”, “poor supervision” and “social pressures”. There is no doubt that individuals’ beliefs play a notable role in initiation and continuation of cigarette smoking; therefore, the researchers recommend the policy makers and health sciences experts to detect wrong beliefs about smoking among children and replace them with right beliefs as soon as possible.

There is no doubt that the friendly relation between parents and children is a protective factor in the prevention of unhealthy behaviors like smoking. However, parents must be careful that this relationship does not proceed to unhealthy behaviors such as ignoring their children’s smoking. Researchers propose that parents have a good relationship with their children, but beside that, they must be able to prevent and change unhealthy behaviors of their children. The families, in which problems with supervision of the children and the adolescents exist, are severely vulnerable to initiation and continuation of smoking, and possibly, other behavioral problems. Formation of a “comprehensive health service system” in which all the people are under constant care may support this group of families. The findings of the study showed that people probably go toward unhealthy behaviors such as smoking more at the time of social crisis like wars. Awareness of the fact that the initiation of smoking may increase in such times, can enhance their preparedness to face this problem. 

In this study we had to select the participants from acquainted persons due to the lack of strangers’ preparedness to undergo interview and voice recording. Anyhow, we tried to select those acquainted persons who varied concerning their occupation, education, age, and level of dependency to smoking. 

## Conclusion

Smokers’ experiences have an important role in identity of initiation and continuation of smoking. Present study showed smoking may start from childhood, smoking sibling can be dangerous for non-smoking sibling, smoking may increase in happy events as well as sad events, physicians and policy-makers that smoke may normalize smoking for communities, wrong beliefs play a role in smoking, and familial and social stress have important roles in smoking. Better understanding of smokers’ experiences about initiation and continuation of smoking by nurses and other health specialists can be effective on smoking prevention and cessation. 

In this study, a number of predisposing factors for initiation and continuation of smoking were identified, since smoking is a gateway to drug abuse and the majority of drug users were smokers in the past, we propose predisposing factor for drug abuse and how to move from cigarettes to drugs are investigated. 
